# Supplementary material for: An analysis of frailty and multimorbidity in 20,566 UK Biobank participants with type 2 diabetes
Source: Commun Med (Lond). 2021 Aug 27;1:28. doi: 10.1038/s43856-021-00029-9 (PMC9053176; doi:10.1038/s43856-021-00029-9)
Supplement: Supplementary file 1 — Description of Additional Supplementary Files [file 43856_2021_29_MOESM1_ESM.pdf]

Description of Additional Supplementary Files

File Name: Supplementary Data 1

Description: ICD-10 codes for charlson index conditions

File Name: Supplementary Data 2

Description: conditions included in count of long-term conditions (self-reported terms and ICD-10 codes)

File Name: Supplementary Data 3

Description: Frailty index deficits

File Name: Supplementary Data 4

Description: Relationship between frailty and baseline characteristics

File Name: Supplementary Data 5

Description: Relationship between multimorbidity and baseline characteristics

File Name: Supplementary Data 6

Description: Relationship between frailty, multimorbidity, age, sex, and adverse outcomes

File Name: Supplementary Data 7

Description: Data underlying figures within main manuscript text
