# Supplementary material for: An analysis of frailty and multimorbidity in 20,566 UK Biobank participants with type 2 diabetes
Source: Commun Med (Lond). 2021 Aug 27;1:28. doi: 10.1038/s43856-021-00029-9 (PMC9053176; doi:10.1038/s43856-021-00029-9)
Supplement: Supplementary file 2 — Supplementary Materials [file 43856_2021_29_MOESM2_ESM.pdf]

Supplementary material: **An analysis of frailty and multimorbidity in 20,566 UK Biobank participants with Type 2 Diabetes**

Peter Hanlon, Bhautesh D Jani, Elaine Butterly, Barbara Nicholl, Jim Lewsey, David A McAllister, Frances S Mair

Supplementary Tables

Relationship between frailty/multimorbidity and age

Supplementary table 1 Frailty index			
Frailty index value	Age 40-50	Age 50-60	Age 60-72
0-0.05	163 (8.8%)	345 (5.8%)	665 (5.2%)
0.5-1.0	415 (22.3%)	1264 (21.2%)	2739 (21.5%)
1.0-1.5	422 (22.7%)	1463 (24.6%)	3330 (26.1%)
1.5-2.0	361 (19.4%)	1152 (19.4%)	2683 (21%)
>2.0	497 (26.7%)	1729 (29%)	3338 (26.2%)

This table shows the number of participants of each level of frailty index, stratified by age. Percentages in brackets show the prevalence within age strata.

Supplementary table 2 Frailty phenotype			
Frailty phenotype	Age 40-50	Age 50-60	Age 60-72
Robust	474 (25.5%)	1713 (28.8%)	4074 (31.9%)
Pre-frail	1082 (58.2%)	3180 (53.4%)	6764 (53%)
Frail	235 (12.6%)	797 (13.4%)	1473 (11.5%)
NA	67 (3.6%)	263 (4.4%)	444 (3.5%)
Individual components			
Weight loss	672 (36.2%)	1935 (32.5%)	3455 (27.1%)
Low grip strength	370 (19.9%)	1531 (25.7%)	4070 (31.9%)
Low physical activity	379 (20.4%)	1160 (19.5%)	1968 (15.4%)
Exhaustion	543 (29.2%)	1482 (24.9%)	2051 (16.1%)
Slow walking pace	366 (19.7%)	1313 (22.1%)	3055 (24.0%)

This table shows the number of participants of each level of frailty phenotype, stratified by age. Percentages in brackets show the prevalence within age strata. The number (and percentage) of participants with each of the individual components of the frailty phenotype is also shown, stratified by age.

Supplementary table 3 Charlson Index			
Value	Age 40-50	Age 50-60	Age 60-72
0	1335 (71.9%)	3897 (65.5%)	6826 (53.5%)
1	380 (20.5%)	1365 (22.9%)	3297 (25.8%)
2-13	143 (7.7%)	691 (11.6%)	2632 (20.6%)

This table shows the number of participants of each level of charlson index, stratified by age. Percentages in brackets show the prevalence within age strata.

Supplementary table 4 LTC count			
Count	Age 40-50	Age 50-60	Age 60-72
0	381 (20.5%)	761 (12.8%)	950 (7.4%)
1	600 (32.3%)	1726 (29%)	3131 (24.5%)
2	380 (20.5%)	1454 (24.4%)	3281 (25.7%)
3	234 (12.6%)	903 (15.2%)	2387 (18.7%)
4 or more	263 (14.2%)	1109 (18.6%)	3006 (23.6%)

This table shows the number of participants of each level of the count of long-term conditions, stratified by age. Percentages in brackets show the prevalence within age strata.

Supplementary figures

Supplementary figure 1: Correlation between measures

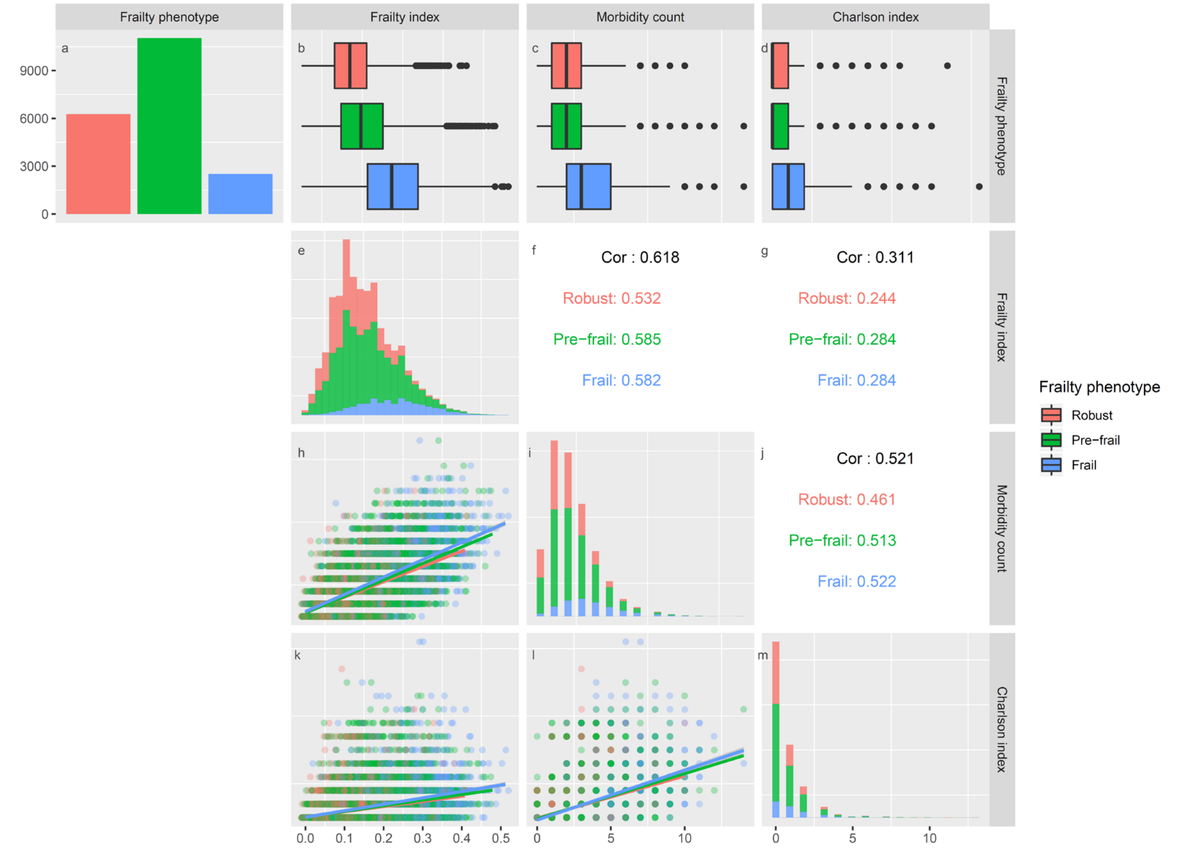

This plot shows the distribution of each measure of frailty or multimorbidity, as well as the correlation between each of the measures. Panel a shows the distribution of the frailty phenotype. Panels e, i, and m show the distribution of the frailty index, long-term condition count, and charlson index, respectively, with the corresponding frailty phenotype levels shown in colour. Box-plots in pabels b, c, and d show the median, interquartile range, range, and outliers of the frailty index, long-term condition count, and charlson index, respectively, stratified by levels of the frailty phenotype. Scatter plots in panels h, k and l show the correlation between the frailty index and the long term condition count (panel h, with correlation coefficients shown in panel f), the frailty index and the charlson index (panel k, with correlation coefficients shown in panel g) and the long-term condition count and the charlson index (panel l, with correlation coefficients shown in panel j). Correlation coefficients are shown for all participants (black text) and stratified by level of the frailty phenotype (coloured text).

Supplementary figure 2: Relationship between HbA1c and mortality, stratified by baseline use of insulin or sulphonylurea

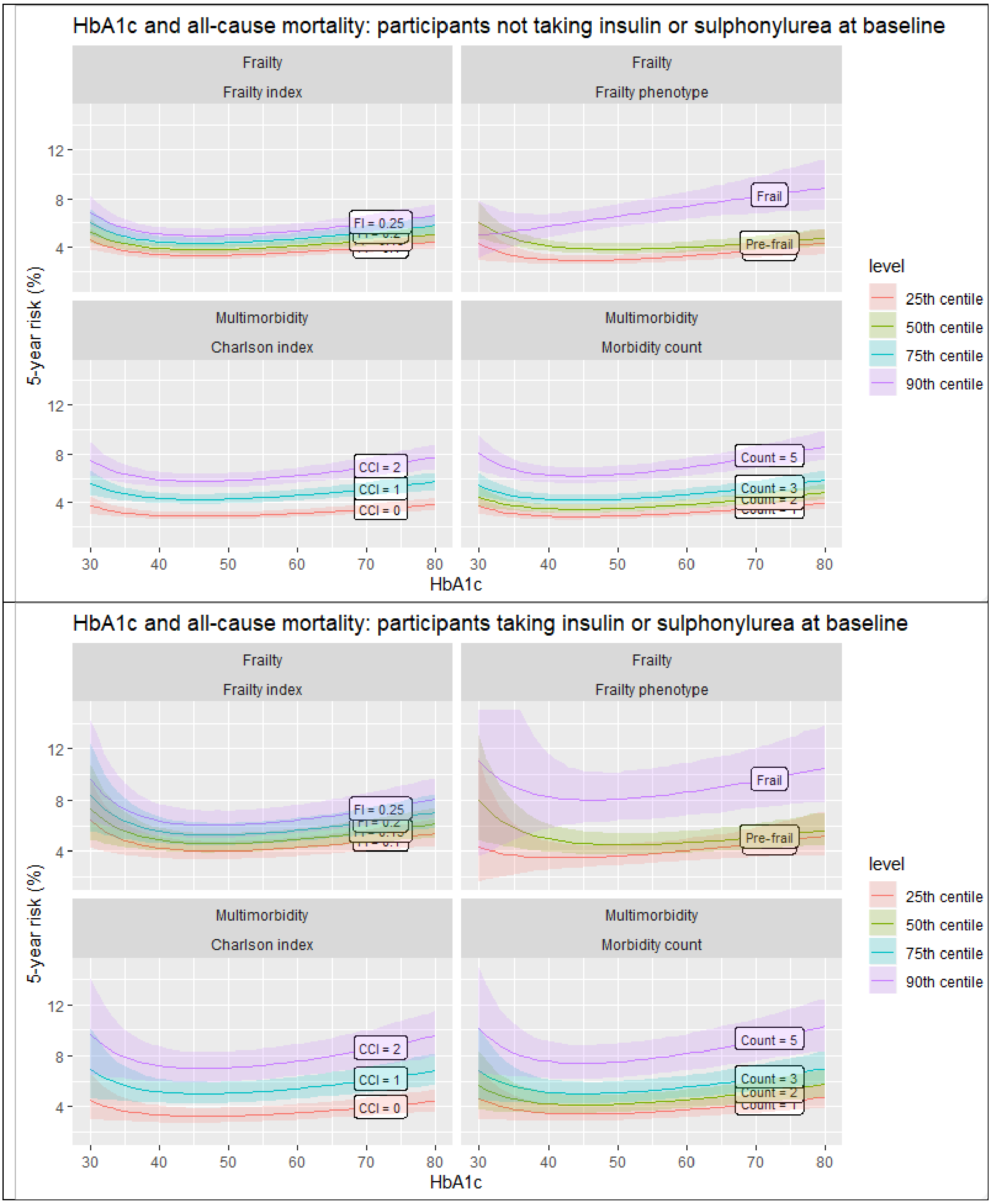

This figure shows the relationship between HbA1c and predicted 5-year mortality at different levels of frailty or multimorbidity, stratified by baseline use of hypoglycaemic agents (insulin or sulphonylurea). Frailty of multimorbidity measure is indicated by the panel labels. Coloured lines or points indicate point estimates for predicted 5-year mortality. Colours indicate the level of frailty or multimorbidity according to centiles. Shaded areas indicate 95% confidence intervals. Models are adjusted for age, sex, socioeconomic status, body mass index, smoking and alcohol. Predicted 5-year mortality is based on age 60, socioeconomic status and body mass index held at the sample mean, previous smokers, and 1-4 times weekly. There was a significant interaction between the frailty

phenotype and HbA1c. Interactions between frailty index, Charlson index, and LTC count were not significant.
