# Supplementary material for: An analysis of frailty and multimorbidity in 20,566 UK Biobank participants with type 2 diabetes
Source: Commun Med (Lond). 2021 Aug 27;1:28. doi: 10.1038/s43856-021-00029-9 (PMC9053176; doi:10.1038/s43856-021-00029-9)
Supplement: Supplementary file 11 — Reporting Summary [file 43856_2021_29_MOESM11_ESM.pdf]

nature research

Corresponding author(s): Peter Hanlon

Last updated by author(s): Jan 29, 2021

Reporting Summary

Nature Research wishes to improve the reproducibility of the work that we publish. This form provides structure for consistency and transparency in reporting. For further information on Nature Research policies, see our Editorial Policies and the Editorial Policy Checklist.

Statistics

For all statistical analyses, confirm that the following items are present in the figure legend, table legend, main text, or Methods section.

n/a	Confirmed	
☐	☒	The exact sample size (*n*) for each experimental group/condition, given as a discrete number and unit of measurement
☐	☒	A statement on whether measurements were taken from distinct samples or whether the same sample was measured repeatedly
☐	☒	The statistical test(s) used AND whether they are one- or two-sided<br>*Only common tests should be described solely by name; describe more complex techniques in the Methods section.*
☐	☒	A description of all covariates tested
☐	☒	A description of any assumptions or corrections, such as tests of normality and adjustment for multiple comparisons
☐	☒	A full description of the statistical parameters including central tendency (e.g. means) or other basic estimates (e.g. regression coefficient) AND variation (e.g. standard deviation) or associated estimates of uncertainty (e.g. confidence intervals)
☒	☐	For null hypothesis testing, the test statistic (e.g. *F*, *t*, *r*) with confidence intervals, effect sizes, degrees of freedom and *P* value noted<br>*Give P values as exact values whenever suitable.*
☒	☐	For Bayesian analysis, information on the choice of priors and Markov chain Monte Carlo settings
☒	☐	For hierarchical and complex designs, identification of the appropriate level for tests and full reporting of outcomes
☒	☐	Estimates of effect sizes (e.g. Cohen's *d*, Pearson's *r*), indicating how they were calculated

Our web collection on statistics for biologists contains articles on many of the points above.

Software and code

Policy information about availability of computer code

Data collection	Data were supplied from UK Biobank access management system
Data analysis	All data processing and statistical analyses were performed using R.

For manuscripts utilizing custom algorithms or software that are central to the research but not yet described in published literature, software must be made available to editors and reviewers. We strongly encourage code deposition in a community repository (e.g. GitHub). See the Nature Research guidelines for submitting code & software for further information.

Data

Policy information about availability of data

All manuscripts must include a data availability statement. This statement should provide the following information, where applicable:

- Accession codes, unique identifiers, or web links for publicly available datasets
- A list of figures that have associated raw data
- A description of any restrictions on data availability

All participant data are held by UK Biobank and are available on application to UK Biobank. All code used to generate variables and analyses will be returned to UK Biobank for record and will be available to third party researchers.

Field-specific reporting

Please select the one below that is the best fit for your research. If you are not sure, read the appropriate sections before making your selection.

☒ Life sciences ☐ Behavioural & social sciences ☐ Ecological, evolutionary & environmental sciences

For a reference copy of the document with all sections, see nature.com/documents/nr-reporting-summary-flat.pdf

Life sciences study design

All studies must disclose on these points even when the disclosure is negative.

Sample size	All UK Biobank participants with type 2 diabetes at baseline.
Data exclusions	Complete case analysis for adjusted regression models (missing data detailed in supplementary material).
Replication	All code used to generate the variables and analyses will be returned to UK Biobank from whom it will be available to third party researchers.
Randomization	Observational study comparing levels of frailty/multimorbidity. Analysis groups were allocated based on baseline characteristics. Analyses adjusted for age, sex, socioeconomic status, smoking and alcohol.
Blinding	Not applicable.

Reporting for specific materials, systems and methods

We require information from authors about some types of materials, experimental systems and methods used in many studies. Here, indicate whether each material, system or method listed is relevant to your study. If you are not sure if a list item applies to your research, read the appropriate section before selecting a response.

Materials & experimental systems

n/a	Involved in the study	
☒	☐	Antibodies
☒	☐	Eukaryotic cell lines
☒	☐	Palaeontology and archaeology
☒	☐	Animals and other organisms
☐	☒	Human research participants
☒	☐	Clinical data
☒	☐	Dual use research of concern

Methods

n/a	Involved in the study	
☒	☐	ChIP-seq
☒	☐	Flow cytometry
☒	☐	MRI-based neuroimaging

Human research participants

Policy information about [studies involving human research participants]()

Population characteristics	Participants were volunteers to the UK Biobank research cohort (age 40-70) who were identified at baseline to have type 2 diabetes according to a previously validated algorithm. Mean age 60.2. 38.6% female in the type 2 diabetes sample.
Recruitment	Participants were recruited between 2006-2010 by postal invitation and attended one of 22 assessment centers in England, Scotland or Wales where they completed a touchscreen questionnaire, a nurse interview, had physical measurements, and provided blood samples.
Ethics oversight	The UK Biobank has full ethical approval from the NHS National Research Ethics Service (16/NW/0274). This work was conducted under project 14151.

Note that full information on the approval of the study protocol must also be provided in the manuscript.
